# A new modelling approach for predicting process evolution of cork-rubber composites slabs vulcanization

**DOI:** 10.1038/s41598-022-11849-7

**Published:** 2022-05-14

**Authors:** Helena Lopes, Susana P. Silva, João Paulo Carvalho, José Machado

**Affiliations:** 1 MEtRICs Research Center, University of Minho, Campus of Azurém, 4800-058 Guimarães, Portugal; 2 Amorim Cork Composites, Rua Comendador Américo Ferreira Amorim, 260, 4535-186 Mozelos VFR, Portugal

**Keywords:** Engineering, Mechanical engineering

## Abstract

In order to predict the evolution of the vulcanization process of cork-rubber slabs, a numerical approach was developed combining heat transfer by conduction and kinetics models. A one-dimensional model was applied to predict the evolution of temperature and degree of cure at different stages of the vulcanization of a cork-rubber composite. Also, due to the degradation verified by the compound, an existent reversion model was added to the problem. Based on rheometer data, cure and reversion parameters were determined. Experimental data were used to determine the thermal properties of the compound, assuming a constant value or according to its degree of cure and temperature. The results obtained by simulation showed a good correspondence with experimental results, even when assuming constant thermal properties. The application of the proposed methodology provides information about the optimum process parameters for each thickness slab, without compromising the homogeneity and characteristics of the final product, which can be a valuable tool during the development and product stages of cork-rubber composites.

## Introduction

Cork-rubber composites are elastomeric materials composed of a rubber matrix filled with cork granules. The presence of cork contributes to the improvement of recovery and to the reduction of lateral flow, due to its low Poisson’s ratio, when the composite is submitted to compressive loads. Also, it provides the chemical stability of the rubber mixture. Depending on the type of rubber, these composites have good fluid resistance makes them suitable for applications like gaskets used in the automotive or power industries, for example. Other applications of cork-rubber composites include vibration or acoustic isolation purposes, in form of pads. The manufacture of cork-rubber composite materials is similar to other rubber compounds^1,2^. To a rubber compound formulation, cork granules are added during mixing stages, along with other raw materials like the vulcanizing agent, vulcanization activators, reinforcing fillers, process aids, anti-degradants, among others. After passing by a two-roll mill to achieve a homogeneous mixture and a constant thickness slab, the material is placed inside a metallic mold and vulcanized in a compression molding press.

The vulcanization process results from a combination of two phenomena: heat transfer and curing reaction^3^. This stage is considered a crucial step towards achieving the optimum final characteristics of a rubber product. During vulcanization, crosslinks between polymeric chains are created, generally with the release of energy, transforming material’s properties^4^. The physical and mechanical properties of rubber products are very related to the vulcanization parameters, as reported by other authors^5,6^.

To control the evolution of curing reactions, different methods can be applied including differential scanning calorimetry (DSC), oscillating disc rheometer (ODR), dynamic mechanical analysis (DMA), moving die rheometer (MDR) and even dielectric analysis (DEA)^7^. In terms of rheology, the shear modulus of a rubber compound increases as new crosslinks are created. An MDR test provides information about the evolution of the degree of cure of a thin disk sample when subjected to an isothermal process, through the continuous measurement of torque of one of the dies^8^. Rheology of a rubber compound can also give information about the optimal curing parameters, like temperature and time of vulcanization since these are influential variables in a vulcanization process.

However, achieving the expected characteristics of rubber products can be challenging, due to its geometry, compound characteristics and other aspects of the manufacturing process. The application of modelling approaches as a tool in the aid to optimize rubber vulcanization has been studied by several authors. Generally, these works present a methodology combining cure with the heating problem including model-based^9–11^ and data-driven approaches^12,13^.

Regarding the first methodology approach, several cure kinetics models have been proposed to evaluate the degree of cure of a sample, according to the stage of vulcanization process: induction, curing and post-cure. Phenomenological models, based on the experimental data fitting, are the most common to find, although some mechanistic models are also available in the literature^14^. The work of Ghoreishy^14^ presents a detailed review of several approaches to simulate each stage of the vulcanization process.

In terms of the heat transfer problem, several considerations about the thermal properties have been tested, such as considering density and specific heat as constant values^15^, assuming a temperature and degree of cure dependence of thermal conductivity and specific heat^16,17^, or using values of thermal diffusivity of vulcanized elastomers obtained by experimental and model fitting methods according to their temperature^18^.

The goal of this study was to evaluate the application of a numerical approach combining heat transfer and cure models to simulate the non-isothermal vulcanization process of cork-rubber composite slabs with different thicknesses at different vulcanization temperatures. Using rheometer test results, two phenomenological models were applied to represent curing and reversion stages. In the first case, the model of Isayev and Deng^19^ was chosen and solved following a similar approach presented by Pinheiro^20^. Regarding reversion, the method proposed by Bera et al.^11^ was employed. Besides the results obtained by MDR, to feed the model, a methodology to determine thermal properties was also conducted. The results obtained by the application of the methodology were compared against experimental data for validation purposes. Also, several conditions were introduced in the model to study optimum process parameters according to different thickness slabs.

This article is organized in the following order. In chapter 2 theoretical background behind the model’s development is presented; “Materials and methods” section presents the experimental procedure and simulation methodology followed; in chapter 4 results obtained by both experimental and modelling approaches are analyzed and in “Conclusions” section conclusions are presented.

## Theoretical background

### Heat transfer model

The governing equations of the conduction heat transfer problem, using the Cartesian coordinate system with three and one dimensions, are presented in Eqs. (1) and (2), respectively.

1 $$\frac{1}{\text{a}}\frac{\partial \text{T}}{\partial \text{t}}=\frac{{\partial }^{2}\text{T}}{\partial {\text{x}}^{2}}+\frac{{\partial }^{2}\text{T}}{\partial {\text{y}}^{2}}+\frac{{\partial }^{2}\text{T}}{\partial {\text{z}}^{2}}+\frac{\dot{\text{Q}}}{\lambda },$$

2 $$\frac{1}{\text{a}}\frac{\partial \text{T}}{\partial \text{t}}=\frac{{\partial }^{2}\text{T}}{\partial {\text{x}}^{2}}+\frac{\dot{\text{Q}}}{\lambda },$$

where $T$ is temperature (K), $t$ is time (s), $a$ represents thermal diffusivity (m^2^/s), $\dot{Q}$ is the heat source term (W/m^3^), $\lambda$ is thermal conductivity (W/m/K) and $x, y, z$ correspond to each coordinate (m). Thermal diffusivity is given by Eq. (3).

3 $$\text{a}=\frac{\lambda }{\uprho {\text{c}}_{\text{p}}}.$$

In vulcanization studies, generally, density ($\rho$ in kg/m^3^) is assumed to be constant, although some authors consider the dependence of crosslinking density^14^. The thermal properties of a rubber compound—specific heat (${c}_{p}$ in J/kg/K) and thermal conductivity—can be expressed in terms of the sample’s degree of cure as well as its temperature. The following expressions have been applied to several works^16,17^.

4 $$\lambda =\left(1-\alpha_{\text{c}}\right){\lambda }_{\text{u}}\left(\text{T}\right)+\alpha_{\text{c}}{\lambda }_{\text{v}}\left(\text{T}\right),$$

5 $${\text{c}}_{\text{p}}=\left(1-\alpha_{\text{c}}\right){{\text{c}}_{\text{p}}}_{\text{u}}\left(\text{T}\right)+\alpha_{\text{c}}{{\text{c}}_{\text{p}}}_{\text{v}}\left(\text{T}\right),$$

where ${\alpha }_{c}$ is the degree of cure (–) and subscripts $u$ and $v$ represent the uncured and cured samples, respectively.

In this study, the heat source term is related to the curing reaction since it is an exothermic process. The heat source term related to a vulcanization reaction can be expressed in terms of rate of cure, as stated in Eq. (6).

6 $$\dot{\text{Q}}={\text{Q}}_{\infty }\frac{\text{d}\alpha_{\text{c}}}{\text{dt}},$$

where ${Q}_{\infty }$ is the total heat of the curing reaction (J/m^3^). The degree of cure at a specific time $t$ corresponds to the ratio between the heat generated up to time $t$ (${Q}_{t}$) and total heat of the curing reaction (Eq. (7)).

7 $$\alpha_{\text{c}}=\frac{{\text{Q}}_{\text{t}}}{{\text{Q}}_{\infty }}.$$

### Cure kinetics model

The present study combines the application of two models for curing^19^ and reversion stages^11^. Regarding the curing stage, Isayev and Deng’s model was applied to determine the degree of cure (${\alpha }_{c}$) and rate of cure ($d{\alpha }_{c}/dt$), as presented in Eqs. (8) and (9)^19^.

8 $$\alpha_{\text{c}}=\frac{{\text{k}}_{\text{c}}{\text{t}}^{\text{n}}}{1+{\text{k}}_{\text{c}}{\text{t}}^{\text{n}}},$$

9 $$\frac{\text{d}\alpha_{\text{c}}}{\text{dt}}=\frac{\text{n}}{{\text{k}}_{\text{c}}}{\text{t}}^{-1-\text{n}}\alpha_{\text{c}}^{2},$$

where ${k}_{c}$ and $n$ are rate constant (s^−1^) and order of reaction, respectively.

Bera et al.^11^ proposed a methodology to determine the degree (${\alpha }_{r}$) and rate ($d{\alpha }_{r}/dt$) associated with the reversion stage. The expressions presented in Eqs. (10) and (11) include a temperature-dependent variable, $x$, that can be calculated based on rheometer data, as explained in the following sections of the article.

10 $$\alpha_{\text{r}}=\text{x}\left(1-{\text{e}}^{-{\text{k}}_{\text{r}}\text{t}}\right),$$

11 $$\frac{\text{d}\alpha_{\text{r}}}{\text{dt}}={\text{k}}_{\text{r}}\left(\text{x}-\alpha_{\text{r}}\right),$$

where ${k}_{r}$ is the value of the rate constant associated with the reversion stage (s^−1^). This variable can be determined using on Arrhenius equation (Eq. (12)). The determination of activation energy ${E}_{{a}_{r}}$ (J/mol) and pre-exponential factor ${A}_{r}$ (s^−1^) related to the reversion stage are presented in the following sections.

12 $${\text{k}}_{\text{r}}={\text{A}}_{\text{r}}{\text{e}}^{-\frac{{{\text{E}}_{\text{a}}}_{\text{r}}}{\text{RT}}}.$$

$R$ = 8.314 J/K/mol is the universal gas constant.

The approach presented by Bera et al.^11^ also includes the concept of a combined degree of cure, also designated by vulcanization degree ($\alpha$), that can be obtained indirectly by subtracting the terms relative to the rate of curing and reversion stages, as indicated by Eq. (13).

13 $$\frac{\text{d}}\alpha {\text{dt}}=\frac{\text{d}\alpha_{\text{c}}}{\text{dt}}-\frac{\text{d}\alpha_{\text{r}}}{\text{dt}}.$$

### Moving die rheometer

**Figure 1:**
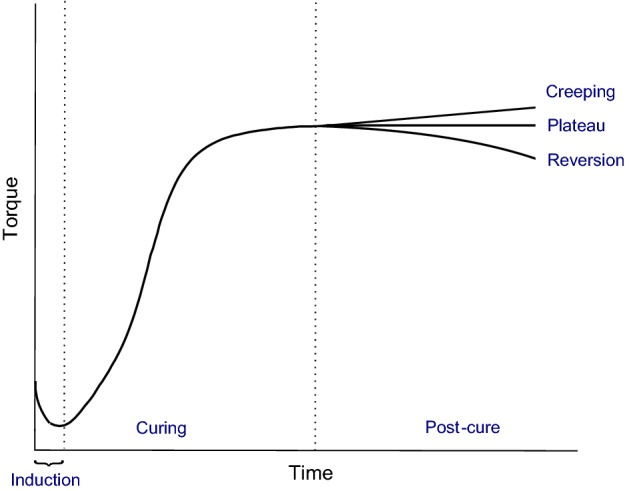
Different stages of a rheometer curve.

A moving die rheometer (MDR) test provides information about the evolution of the degree of cure of a thin disk sample placed between two dies subjected to an isothermal process, through the continuous measurement of torque of one of the dies^8^. A typical rheometer curve can be divided into three stages: induction, curing and post-cure, as indicated in Fig. 1. The post-cure phase can present different behaviors according to the rubber compound. The compound can increase its stiffness, stabilize or begin to degrade as it happens with natural rubber^4^.

During the curing stage, information about the degree of cure of the sample at each time point can be determined from the vulcanization curve (according to Eq. (14))^14^:

14 $$\alpha_{\text{c}}=\frac{{\text{M}}_{\text{t}}-{\text{M}}_{\text{L}}}{{\text{M}}_{\text{H}}-{\text{M}}_{\text{L}}},$$

where ${M}_{t}$ represents torque at time $t$ (dNm) and ${M}_{L}$ and ${M}_{H}$ represent the lowest and highest torque registered in the rheometer test, respectively.

Different vulcanization temperatures present different curves. Assuming that the curing reaction has first-order kinetics, based on the Arrhenius equation, a relation between the data obtained at two different temperatures (${T}_{r}$ and ${T}_{1}$) can be determined^12^. This method is designated by equivalent cure concept (ECC), presented in Eq. (15).

15 $${\text{t}}_{\text{r}}={\text{t}}_{1}{\text{e}}^{\frac{{\text{E}}_{\text{a}}}{\text{R}}\left(\frac{1}{{\text{T}}_{\text{r}}}-\frac{1}{{\text{T}}_{1}}\right)},$$

where ${E}_{a}$ is the activation energy (J/mol), ${t}_{r}$ and ${t}_{1}$ represent time (s) correspondent to the same degree of cure concerning vulcanization temperature ${T}_{r}$ and ${T}_{1}$, respectively.

## Materials and methods

### Experimental tests

**Figure 2:**
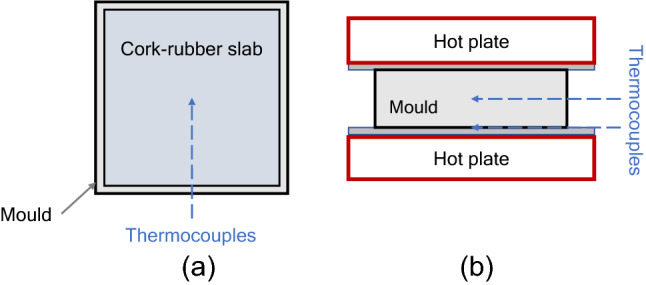
Compression molding: (**a**) mold and cork rubber slab; (**b**) representation of the vulcanization process.

The cork-natural rubber compounds used in this study were given by a cork-rubber composites supplier. These compounds use sulfur as the vulcanizing agent, and vulcanizates present a hardness range between 50 and 60 Shore A. A two-roll open mill was used to obtain slabs of uniform thickness. After conducting isothermal rheometer tests at different temperatures from 140 to 180 °C, samples were cut in square shapes of 200 × 200 mm. Layers of the same material were piled up together to obtain the mass necessary to obtain a final 10 mm or 30 mm thickness cork-rubber vulcanizate. Based on information about the cork-rubber compound given by the material’s supplier, vulcanization times were defined. At higher vulcanization temperatures, vulcanization time was prolonged until the sample’s center achieved the vulcanization temperature, to evaluate also reversion stage. To evaluate the temperature profile in the center during compression molding, a thermocouple was inserted in the middle of the sample. Another thermocouple was placed between the lower surface of the rubber sample and the inferior hot plate. Four samples were vulcanized, and their temperature was monitored throughout the vulcanization process: two 10 mm samples vulcanized at 150 °C and 180 °C, and other two 30 mm samples vulcanized at the same temperatures. A scheme of the compression molding process is presented in Fig. 2.

Another experience was performed to determine the thermal conductivity of a vulcanized sample. Using an heating chamber, one of the produced samples was heated from ambient temperature to 185 °C. The temperature evolution at the surface and center of the sample was recorded applying the same thermocouple method. The same equipment was applied in all experimental tests (compression molding and heating chamber setups) with a total thermocouple measurement error of ± 2.7 °C.

### Determine cork-rubber compound properties

#### Thermal conductivity and diffusivity

Using temperature data obtained during the production of all samples, an approximate solution to the analytical problem of transient heat transfer in a one-dimensional slab was performed, as presented in the work of Bafrnec et al.^21^. Considering that the studied curing reaction had a low impact in the temperature variation at the center of a slab, finite differences method, using the explicit form, was applied to solve the conduction heat transfer equation (Eq. (2)). To determine the thermal diffusivity, it was considered a constant value of the product between specific heat and density, as assumed in the work of Prentice and Williams^15^. Thermal diffusivity was continuously updated, through change of thermal conductivity value, until achieving a minimum error between observed and predicted results, applying the generalized reduced gradient (GRG) procedure available in Solver add-in of Excel spreadsheet (see^22,23^ for more details about the algorithm).

As a first approach to estimate a value for thermal diffusivity and conductivity, all data obtained throughout the whole vulcanization process was used, to determine a constant value of this parameter.

A second approach considered was dividing the data in temperature intervals of 20 °C, from ambient temperature to 180 °C, until the beginning of the curing reaction in the middle of the slab and applying the previous procedure at each temperature interval. The variation of density and specific heat with temperature was also not considered. This approach results in the variation of thermal conductivity of an uncured cork-rubber composite compound with temperature. The same procedure was applied to the data obtained by the cured sample’s heating in the chamber, to determine the variation of the thermal conductivity of the vulcanizate with temperature.

#### Reversion parameters

Since reversion occurs in natural rubber compounds, such as the ones studied in this work, the reversion stage was also studied. The parameters related to the reversion stage of the cork-rubber compound were determined based on the method proposed by Bera et al.^11^. Rheometer data obtained at different vulcanization temperatures (150, 160, 170 and 180 °C), containing information about the reversion phase, were used to determine the parameters presented in “Cure kinetics model” section. For each temperature, ${k}_{r}$ and $x$ were determined by minimizing the squared error between experimental and predicted torque by the method of Bera et al.^11^, using the GRG procedure available in Solver add-in of Excel. The same approach was performed to determine ${A}_{r}$ and ${{E}_{a}}_{r}$ in the relation between ${k}_{r}$ and vulcanization temperature. The obtained relationship between temperature and $x$ has the following form and boundary restrictions:

16 $$\text{x}={\text{m}}_{\text{x}}\text{T}+{\text{b}}_{\text{x}} , 0<\text{x}<1,$$

17 $$\text{x}=0 ,\text{ x}\le 0,$$

18 $$\text{x}=1 ,\text{ x}\ge 1,$$

where ${m}_{x}$ and ${b}_{x}$ are slope and y-intercept values, respectively.

### Model

A combined unidimensional heat conduction and kinetics model was used to determine temperature and degree of cure profiles at different locations of a uniform thickness slab. The temperature profile at the surface of the hot plate during the vulcanization process was used as a boundary condition and thermal resistance between plate and cork-rubber sample was not considered. Regarding thermal properties, two approaches were tested: assume a constant value of thermal diffusivity throughout the whole vulcanization process or make it dependent on temperature. Using the latter method, a constant product of density and specific heat was considered, while thermal conductivity was determined as a function of temperature. Vulcanization related properties were obtained by the rheometer testing results. The activation energy was determined based on the Arrhenius plot. At the beginning of the simulations, it was considered that the initial degree of cure was zero.

#### Determine temperature and degree of cure

The first variables to be updated on the proposed model are related to the kinetics model. To determine their values at each location of the slab, the following algorithm was applied, based on the work of Pinheiro^20^:

1. In the first step of the solving algorithm, two points from the rheometer curve of a reference temperature are selected. In this study, times correspondent to 10% and 90% degree of cure, using a vulcanization temperature of 140 °C, were chosen.
2. Applying ECC (Eq. (15)) at each spatial point, the equivalent times (${t}_{e{q}_{1}}$ and ${t}_{e{q}_{2}}$) corresponding to degrees of cure of 10% and 90% and temperature at previous time step (${T}_{m}^{p}$), were calculated.
3. For each spatial point, $n$ and ${k}_{c}$ values can be determined using Eqs. (19) and (20):
  19 $$\text{n}=\frac{\text{ln}\left(\frac{{\alpha_{\text{c}}}_{1}\left(1-{\alpha_{\text{c}}}_{2}\right)}{{\alpha_{\text{c}}}_{2}\left(1-{\alpha_{\text{c}}}_{1}\right)}\right)}{\text{ln}\left(\frac{{\text{t}}_{{\text{eq}}_{1}}}{{\text{t}}_{{\text{eq}}_{2}}}\right)},$$
  20 $${\text{k}}_{\text{c}}=\frac{\left(\frac{{\alpha_{\text{c}}}_{1}}{1-{\alpha_{\text{c}}}_{1}}\right)}{{{\text{t}}_{{\text{eq}}_{1}}}^{\text{n}}}.$$
4. Using the degree of cure value related to time step $p$, its corresponding time can be calculated from Eq. (8).
5. Adding the time increment ($\Delta t$) to the obtained value, degree of cure (${\alpha }_{c}$) and rate of cure $\left(d{\alpha }_{c}/dt\right)$, at present time step $p+1$ can also be calculated using Isayev and Deng’s model^19^ (Eq. (9)).
6. The heat source term ($\dot{Q}$) can then be determined through Eq. (6) and used to calculate the new temperature at each location.

To compute the temperature values at each location, the explicit form of the finite difference method was applied (Eq. (21)), respecting the stability criterion for the unidimensional case (Eq. (22)).

21 $${\text{T}}_{\text{m}}^{\text{p}+1}=\frac{\text{a}\Delta \text{t}}{{\left(\Delta \text{x}\right)}^{2}}\left({\text{T}}_{\text{m}+1}^{\text{p}}+{\text{T}}_{\text{m}-1}^{\text{p}}-2{\text{T}}_{\text{m}}^{\text{p}}+\frac{\dot{\text{Q}}{\left(\Delta \text{x}\right)}^{2}}{\lambda }\right)+{\text{T}}_{\text{m}}^{\text{p}}$$

22 $$\text{Fo}=\frac{\text{a}\Delta \text{t}}{{\left(\Delta \text{x}\right)}^{2}}\le 0.5,$$

where $m$ is the actual point, $p$ corresponds to the previous time step, $\Delta x$ is the spatial step size and $Fo$ represents Fourier number. Considering the temperature dependence, linear interpolation was used to calculate the thermal conductivity of the uncured and cured samples at the temperature of the previous time step. According to the degree of cure and temperature at this time step, the value of thermal conductivity is updated according to Eq. (4). The value of thermal diffusivity is also calculated based on the new value for thermal conductivity.

#### Detect reversion stage

Based on the work of Bera et al.^11^, a reversion model was added to this study in order to provide an indication of the beginning of the reversion stage at each location of the slab. At each time step, the rate constant associated with the reversion stage ($${k}_{r}$$) and temperature-dependent variable ($$x)$$ were calculated using Eqs. (12) and (16). Then, applying Eqs. (10) and (11), the degree and rate of cure associated with the reversion stage can be calculated, as well as the combined degree of cure (vulcanization degree) expressed in Eq. (23):

23 $$\alpha^{\text{p}+1}=\Delta \text{t}{\frac{\text{d}}\alpha {\text{dt}}}^{\text{p}+1}+\alpha^{\text{p}}=\Delta \text{t}\left({\frac{\text{d}\alpha_{\text{c}}}{\text{dt}}}^{\text{p}+1}-{\frac{\text{d}\alpha_{\text{r}}}{\text{dt}}}^{\text{p}+1}\right)+\alpha^{\text{p}}.$$

## Results and discussion

### Experimental results

**Figure 3:**
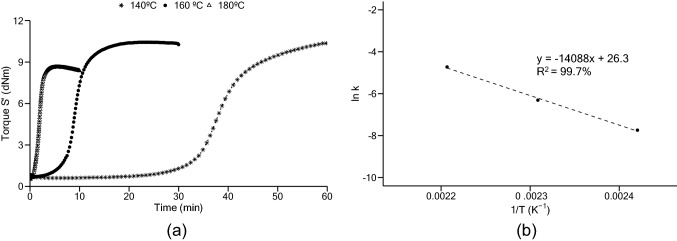
Compound’s cure: (**a**) rheometer data; (**b**) Arrhenius plot.

Rheometer curves of the studied cork-rubber compound are presented in Fig. 3a. Using the data provided by the rheometer test and applying the ECC, presented in Eq. (15), the obtained value for activation energy was 117.13 kJ/mol, considering a degree of cure of 50%. The corresponding Arrhenius plot is shown in Fig. 3b. Vulcanization at higher temperatures produced samples with lower stiffness and higher reversion, probably related to a lower crosslink density, as observed in other works^5,24^.

**Figure 4:**
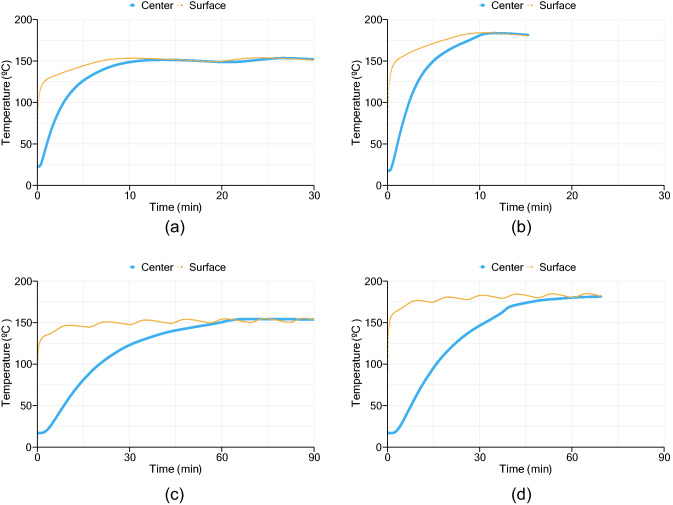
Temperature profiles from all four samples: (**a**) 10 mm sample vulcanized at 150 °C; (**b**) 10 mm sample vulcanized at 180 °C; (**c**) 30 mm sample vulcanized at 150 °C; (**d**) 30 mm sample vulcanized at 180 °C.

Regarding the compression molding process, the temperature evolution registered for each sample during vulcanization is presented in Fig. 4. Each graph shows the temperature values at the center of the slab and between one of the material’s surface and hot plate. Regarding the low thickness samples, it is possible to notice a smooth increase of temperature above 170 °C for the sample vulcanized at 180 °C. The data obtained from the 10 mm sample vulcanized at 150 °C does not show the same behavior, probably due to the low magnitude of energy released from the curing reaction be overlapped with the fluctuation due to the press temperature controller. An increase of temperature due to energy release is also depicted in the center temperature profiles of 30 mm thickness samples. An approximation between surface and center temperatures due to rubber curing reaction, was also reported by Juma and Bafrnec^3^.

### Estimation of thermal conductivity

Recurring to the experimental data obtained at the center of the sample during the vulcanization process, values of thermal conductivity were determined. A density of 1075 kg/m^3^ and a specific heat of 1400 J/kg/K were considered for calculation. In a first approach, using all the experimental data collected from each sample, the mean value obtained for thermal conductivity was 0.1447 W/m/K.

**Figure 5:**
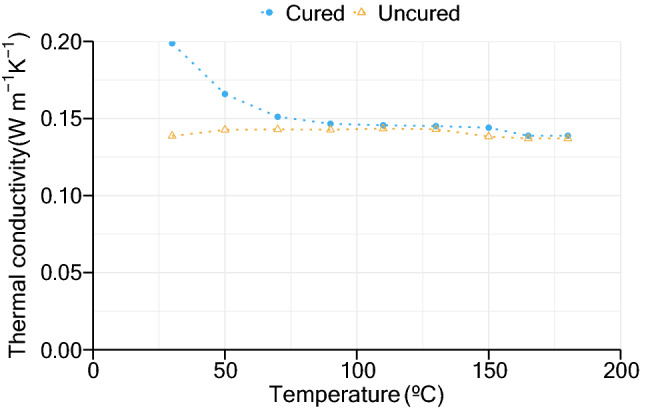
Thermal conductivity of uncured and cured cork-rubber samples.

Another approach was considering the dependence of temperature on the thermal conductivity and degree of cure on uncured and cured cork-rubber samples. The thermal conductivity results obtained are presented in Fig. 5. It was considered that the thermal conductivity remained constant for temperatures above 165 °C.

### Reversion stage

Previous rheometer data from the studied cork-rubber compound containing information about the reversion stage were used as input to determine the values of ${k}_{r}$ and $x$ for each vulcanization temperature. For each of these parameters, curve fitting was applied to determine the correspondent values of ${A}_{r}$ and ${{E}_{a}}_{r}$ as well as the relationship between the parameter $x$ and temperature, expressed in Kelvin. Tables 1 and 2 presents the results regarding the reversion stage.

**Table 1:** Reversion parameters of the studied cork-rubber compound.

Temperature (°C)	Curve fitting—rheometer data		${k}_{r}$(s^−1^)	$x$
	R^2^ (%)	MAPE (%)*		
150	97.7	0.1	2.06 × 10^–4^	0.244
160	99.4	0.2	2.82 × 10^–4^	0.485
170	99.7	0.2	3.04 × 10^–4^	0.771
180	99.4	0.2	4.99 × 10^–4^	0.850

*MAPE* mean absolute percentage error.

**Table 2:** Fitting parameters and results of the applied reversion model.

${k}_{r}$	${A}_{r}$(s^−1^)	${{E}_{a}}_{r}$(J/mol)	R^2^	MAPE
	8380.4	62,644.4	93.3%	14.1%
$x$	${m}_{x}$	${b}_{x}$	R^2^	MAPE
	0.021	− 8.634	95.8%	7.1%

### Simulation of the vulcanization process

The following section presents the results obtained by the application of the presented methodology and its comparison with the experimental results. Regarding initial and boundary conditions, ambient temperature and surface temperature registered in each experimental test were used as input. The data obtained by the rheometer testing at 140 °C and the reversion parameters presented in “Reversion stage” section were also considered as input for the kinetics model. Both thermal conductivity’s determination approaches were considered. Other inputs conditions considered for simulations are presented in Table 3.

**Table 3:** Inputs for the simulation of the vulcanization process.

Input	Value
Density	1075 kg/m^3^
Specific heat	1400 J/kg/K
Activation energy	117.13 kJ/mol
Total heat of the curing reaction	8000 J/kg
Time increment	2 s
Spatial increment	1.67 mm

#### Temperature profiles

**Figure 6:**
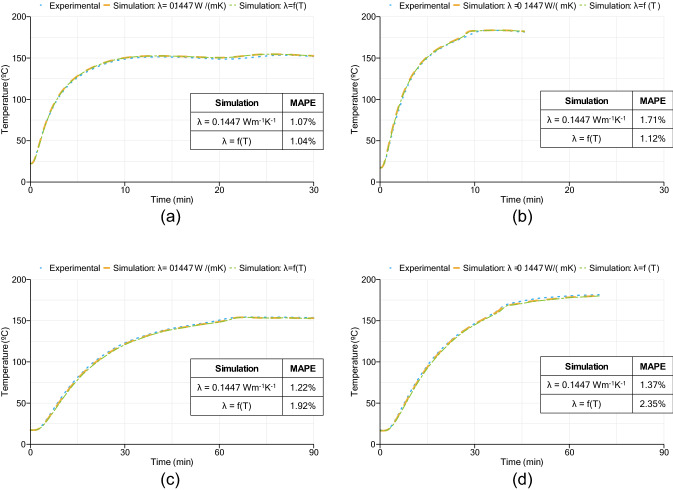
Results of temperature profiles at the center of the slab from experimental and modelling approaches: (**a**) 10 mm sample vulcanized at 150 °C; (**b**) 10 mm sample vulcanized at 180 °C; (**c**) 30 mm sample vulcanized at 150 °C; (**d**) 30 mm sample vulcanized at 180 °C.

Figure 6 presents the temperature profiles at the center of the slab obtained by simulation and experimental methods. Both approaches considering thermal conductivity as constant or as a function of temperature and degree of cure are represented in all graphs. Also, the MAPE values obtained for all simulations are presented.

**Figure 7:**
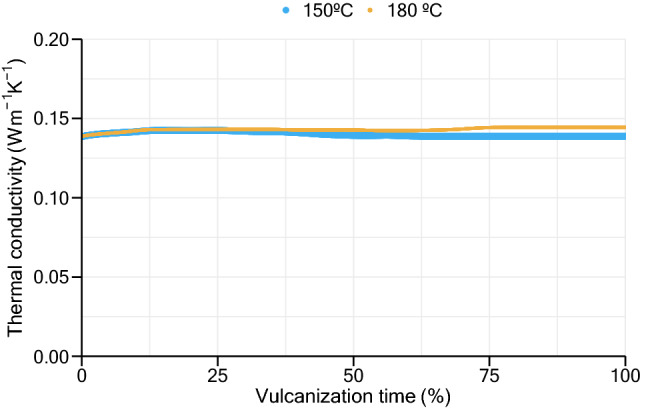
Value of thermal conductivity during the vulcanization process of 30 mm samples vulcanized at 150 °C and 180 °C.

Considering the temperature dependence of thermal conductivity, the maximum error found was 8.6%, related to the simulation of a 30 mm slab vulcanized at 180 °C. Assuming thermal conductivity as a constant value, the maximum error obtained was 9.1% regarding the simulation of a 10 mm slab vulcanized at 180 °C. The MAPE results (below 3%) using both approaches demonstrate a good agreement between simulation and experimental results. Figure 7 shows the evolution of mean thermal conductivity value at each stage of the vulcanization process for the 30 mm slabs vulcanized at 150 °C and 180 °C. As it is possible to observe, thermal conductivity presents a nearly constant behavior throughout the whole vulcanization process. This behavior was also verified in all others simulation cases made for this study. Thus, instead of increasing the complexity of the model, by adding a temperature- and cure-dependent thermal conductivity, a constant value can be used reducing computation time and still providing good proximity with experimental results.

#### Cure evolution

**Figure 8:**
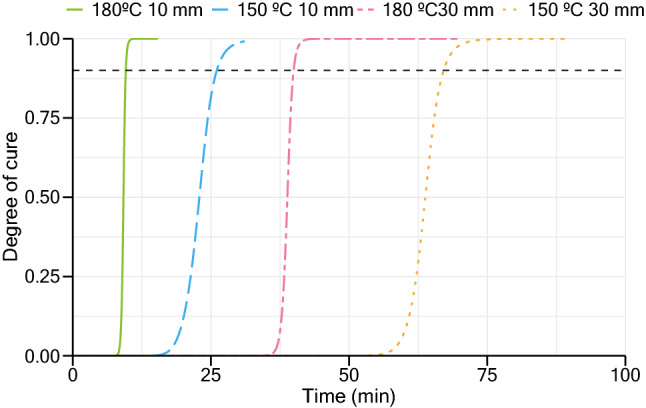
Evolution of the degree of cure at the center of each sample.

Like temperature profiles, an indication of cure evolution at different locations of the sample was also determined. Using the method of constant thermal conductivity, the results of degree of cure at the center of each sample, throughout the process until achieving the maximum degree of cure, are shown in Fig. 8. Simulation results demonstrate that a degree of cure of 90% is obtained in all samples before ending the molding process. All samples produced appeared to be fully cured, and those vulcanized at higher temperatures showed signs of post-cure degradation, such as the decrease of some mechanical properties, darker coloring, appearance of cracks in the sample’s surface and distortion of the final shape.

As expected, and confirmed by rheometer data, vulcanization at high temperature generates an increase in the rate of reaction compared with lower vulcanization temperatures. The increase of slab thickness also indicates a decrease in the rate of cure at the center of the slab. The applied model reproduces these observations as demonstrated by the values of the maximum rate of reaction registered at the center of the slab, for each sample case, presented in Table 4.

**Table 4:** Simulation results of the maximum rate of reaction at the center of each slab.

Vulcanization temperature (°C)	Thickness (mm)	Maximum rate of reaction (s^−1^)
150	10	2.95 × 10^–3^
150	30	2.84 × 10^–3^
180	10	21.85 × 10^–3^
180	30	8.17 × 10^–3^

Applying the reversion model introduced by Bera et al.^11^, an indication of the optimum cure time can be given. In order to have a final product slab with homogenous properties across its thickness, it is important to guarantee the cure of the center of the slab and simultaneously ensure that zones near the surfaces, in contact with the hot plates, do not exhibit clear signs of degradation due to reversion. Taking into consideration the two phenomena, the vulcanization time should validate two conditions, expressed in Eqs. (24) and (25):

24 $$\alpha_{\text{c}}^{\text{center}}\ge 0.9,$$

25 $$\alpha^{\text{surface}}\ge 0.9.$$

**Figure 9:**
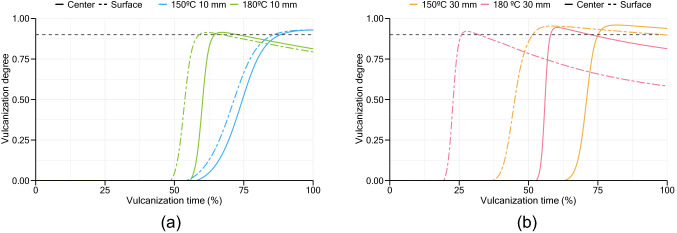
Simulation results for vulcanization degree: (**a**) 10 mm sample; (**b**) 30 mm sample.

In this study, it was considered 90% as the minimum degree of cure to ensure similar properties across the slab. Figure 9 shows the results of vulcanization degree ($\alpha$), combining both cure and reversion models, for 10 mm and 30 mm samples.

**Figure 10:**
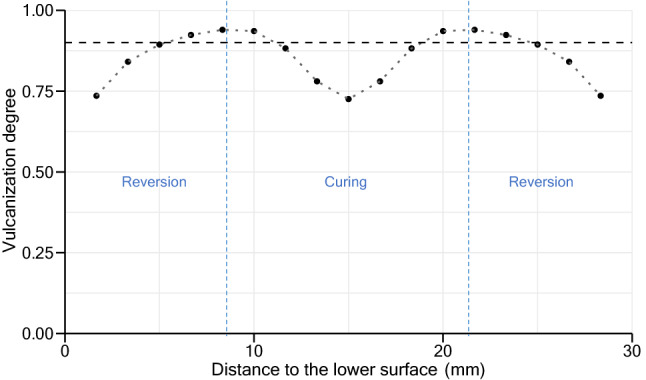
Results of vulcanization degree at different regions of the slab: simulation of 30 mm slab vulcanized at 180 °C.

At higher thicknesses, differences between the degree of cure near to the surfaces and center of the slab are greater than what occurs in lower thickness slabs. When sufficient energy reaches the middle of a thicker slab to start the curing stage, its surfaces have already been subjected to a long period at high temperature compared to lower thickness samples, which enabled them to cure and begin the reversion stage in those areas. Throughout the vulcanization process, at the lowest temperature, differences between the degree of cure at surface and center are lower compared with a vulcanization at 180 °C, resulting in a more homogeneous product across thickness. The intersection between surface and center curves give an indication of the curing time to achieve optimum products, as well as an indication of the minimum vulcanization degree obtained in the produced samples. All samples exhibit a minimum vulcanization degree above or equal to 90%, except the 30 mm sample vulcanized at 180 °C. Results of the vulcanization degree across this sample at the optimum curing time are presented in Fig. 10. As is it possible to verify, the center regions of the slab do not complete their curing stage when other regions near the two hot surfaces are already in the reversion phase.

Based on the simulation approach presented, vulcanization parameters can be determined in order to produce vulcanizates with optimum properties. Regarding the cork-rubber compound studied, the process parameters presented in Table 5 satisfied the conditions presented in both Eqs. (24) and (25). The values presented in Table 5 are in accordance with the process parameters applied by the cork-rubber material supplier.

**Table 5:** Proposal of process parameters to vulcanize the studied cork-rubber material based on simulation results.

Vulcanization temperature (°C)	Thickness (mm)	Min. vulcanization time (min)	Max. vulcanization time (min)
150	10	26	> 31
150	30	67.1	89.2
180	10	9.6	10.3

#### Optimization of process parameters

Another conducted analysis was the determination of the optimum vulcanization times and temperatures according to the product’s thickness. Assuming the same input conditions and a boundary condition with similar behavior to the observed during the experimental procedure, simulations were conducted to predict vulcanization behavior across cork-rubber slabs of several thicknesses—10, 20, 30 and 50 mm—at different temperatures—from 140 to 200 °C.

**Figure 11:**
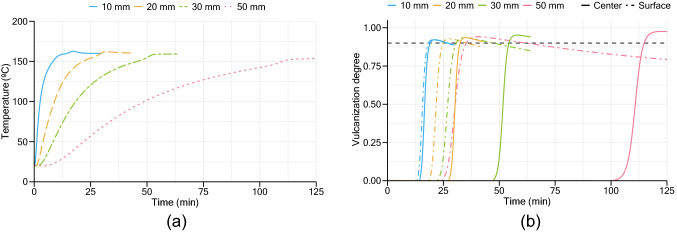
Simulation results of different thickness samples vulcanized at 160 °C: (**a**) center temperature profiles; (**b**) center and near surface’s vulcanization degree.

For example, in a vulcanization at 160 °C, the center’s temperature profiles of each slab obtained by the proposed method are presented in Fig. 11. The vulcanization degree at the center and near to the surfaces of the slab are also both presented in the graph below. Like the experimental results presented in previous sections, an indication of the curing stage at the center is observable in the profiles by a small increase in temperature.

**Figure 12:**
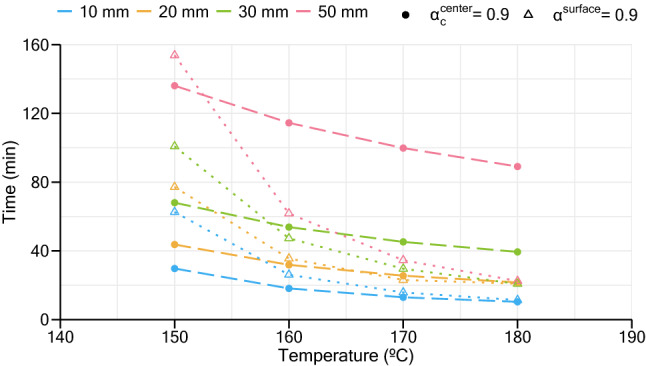
Results of vulcanization times that satisfy Eqs. (24) and (25).

The obtained results regarding the optimum vulcanization parameters are presented in Table 6 and Fig. 12 for all thicknesses. Table 6 presents the vulcanization conditions in which a minimum vulcanization degree of 90% is achieved in the critical regions of the slab: near surfaces and center. It is possible to verify that in order to have optimal properties, the maximum vulcanization temperature allowed decreases as the slab’s thickness increases, to delay the beginning of the reversion stage at locations near to the surfaces and still ensure that the central regions reach a sufficient degree of cure.

**Table 6:** Proposed vulcanization parameters to obtain a minimum vulcanization degree of 90% at the critical regions of the slab.

Thickness (mm)	Max. temperature (°C)	Time (min)
10	196.6	8.1
20	163.2	30.5
30	156.3	59.9
50	151	134.4

Figure 12 presents simulation data for vulcanization temperatures from 150 to 180 °C. A visual representation of the time needed to fulfil a minimum degree of cure 90% at the center, and the moment where surface hits the minimum vulcanization degree of 90% during its reversion stage is presented, showing that the use of low vulcanization temperatures in thicker slabs is critical to obtain homogeneous vulcanizates. Also, the use of lower temperatures increases the time window where vulcanization can occur without compromising the properties of cork-rubber compounds. These results provide information that can be used to aid production management of cork-rubber composites with different dimensions.

For example, consider a 20 mm slab. At 150 °C, a vulcanization time below 44 min is not enough to obtain a fully cured material, while above 77 min the final product can present signs of reversion. Thus, the time difference between obtaining a slab not fully cured and a slab with reversion characteristics is 33 min. With the increase of the vulcanization temperature to 160 °C, this time window reduces to just 4 min. Furthermore, the vulcanization of a 50 mm slab at 150 °C reduces the time frame to 18 min. In this case, with a 10 °C increase in vulcanization temperature, a combination of the two phenomena can be expected since surface reversion occurs before the optimum curing of the slab centre.

## Conclusions

A new methodology developed for the simulation of cork-rubber composites vulcanization is presented, and its application allows the determination of optimum compression molding parameters. Predictions about the evolution of temperature and degree of cure, as well as post-cure behavior, at different locations of a slab, were determined based on the implementation of a numerical methodology combining the heat conduction equation with two kinetics models regarding each stage of the vulcanization of cork-rubber composites: curing and reversion.

Based on information obtained by MDR, curing and reversion parameters were determined by the application of numerical methods. Considering density and specific heat as constant values, thermal conductivity was also determined based on experimentally obtained temperature records following two different approaches: considering or not dependent on temperature and degree of cure.

Simulation results showed good agreement with experimental data of cork-rubber vulcanization at different temperatures and thicknesses, with errors below 10% regarding temperature profiles at the center of the slab.

Differences between the two approaches regarding thermal conductivity were very small since the mean thermal conductivity calculated by the model remained nearly constant and similar to the constant value assumed throughout the vulcanization process. Thus, for the studied cork-rubber composite, the approach considering a constant value of thermal conductivity is validated with the advantage of being the easiest to implement, reducing computation time and still be able to produce results in conformity with experimental data.

Regarding the kinetics study, simulation results allowed to collect information about the optimum vulcanization time, for each temperature and thickness combination. A minimum vulcanization degree parameter (a value of 90% was chosen), combining both effects of cure and reversion, must be achieved to preserve the optimum properties of the compound. Estimates of optimum vulcanization parameters for different samples were determined. Experimental vulcanization times usually applied to produce this cork-rubber compound were validated by the model, with simulations results demonstrating that these values ensure a vulcanization degree above or equal to the optimum value (90%) at all thickness levels of the slab.

Future works include the application of this methodology on different cork-rubber compound formulations. Although it is necessary to feed the model with some experimental data regarding each compound, the application of simulation methods like the one presented provides several advantages. The application of these methodology allows to have a more detailed understanding of the behavior of non-isothermal vulcanization of new cork-rubber composites and obtain information that can be very helpful to optimize development and production stages, by reducing times, energy and material consumption. Also, relating results given by this methodology with the properties of cork-rubber composites could be useful in order to reach the requirements for a final product.

## Data Availability

The data that support the findings of this study are available from Amorim Cork Composites but restrictions apply to the availability of these data, which were used under license for the current study, and so are not publicly available. Data are however available from the authors upon reasonable request and with permission of Amorim Cork Composites.
